# Supplementary material for: The Representation of Children’s Participation in Guidelines for Planning and Designing Public Playspaces: A Scoping Review with “Best Fit” Framework Synthesis
Source: Int J Environ Res Public Health. 2023 May 15;20(10):5823. doi: 10.3390/ijerph20105823 (PMC10218453; doi:10.3390/ijerph20105823)
Supplement: Supplementary file 1 [file ijerph-20-05823-s001.zip › ijerph-2341510-supplementary.pdf]

Supplement File S1: The identification, screening and selection process of the sources.

Identification phase

Four strategies were used to find the intended sources and therefore strengthening the scientific rigor of this scoping review with the gray literature: 1) search in Google, 2) search at the website of play organizations, 3) consultation of experts and 4) citation mining.

Search in Google

With the support of two librarian experts, different search engines and search strings were piloted and established for a search with the words "guidelines design play space" in the search engine Google.

After removing the search histories and checking the settings with the help of an ICT expert, the search in Google search engine was conducted on the 19th of October in Sweden. This resulted in about 388 000 hits. Each URL was opened, and when a document/website was available, the hyperlink was copied to a spreadsheet for the screening process. During this process, Google adapted continuously the number of hits as there seems to run an algorithm for removing duplicates, and after a while the number of URL's were greatly reduced. This process resulted in 203 hyperlinks.

Search at websites of play organizations

The research team consulted the websites of organizations supporting children's play in the community, which they are familiar with. The resource section of the websites was assessed on the availability of a guideline for designing a public playspace. The following websites were assessed in October 2021.

Play Wales	https://www.playwales.org.uk/eng
Play Scotland	https://www.playscotland.org

Play Australia	https://www.playaustralia.org.au
Play Canada	https://playcanada.ca
Play England	https://www.playengland.org.uk
UNICEF	https://www.unicef.org/
Be active kids	http://www.beactivekids.org/
The National Institute for play	http://nifplay.org
Free Play	http://www.freeplaynetwork.org.uk/

This process yielded five documents.

Consultation of experts

The third plan in finding the appropriate sources was approaching experts with a short questionnaire through two organizations known for their network with scholars and practitioners with scientific, policy, management and/or practical expertise in designing a public playspace.

These two organizations were International Play Association and Child in the City, and an email with the following information was sent out with the request to forward it to their members:

Introduction with the relevance, aim and research questions of the study clarifying the reason for sending the request to the person.
1) Are you aware of a guideline for (re)design public playspaces? What is its name?<br>Clarification on concepts used:<br>- Guideline is a document/report for a design team with advice for the design team (not a blog or video clip with advice).

- The advice is about designing a playspace (not about safety or maintenance regulations, not about COVID-19 regulations and not about how children/adults should behave at the playspace).<br>- The guideline is meant to be used in communities or municipalities (not for school or kindergarten playspaces).<br>2) If yes, is there an English version of it?<br>3) Can you share this guideline with the research team for being incorporated in the study? (via link or attachment).
If questions or thoughts will come up regarding your answers, are we allowed to contact you? If so, what is your preferred way?

Eight experts contributed to finding the sources, including two persons from New Zealand, one person from United States of America, one person from Hong Kong, one person from Australia, two persons from Ireland and one person from Scotland, the United Kingdom, which yielded in 130 documents or URL links. This identification strategy resulted in 130 documents or URLs.

Citation mining

The citations of the sources retrieved via the three former search strategies were examined manually. Likewise, the reference list of studies deployed in describing the rationale for this scoping review was examined. This process resulted in zero results.

Screening phase

The identified sources were assessed with the inclusion criteria of an English-language source and describing designing a public playspace. Excluded were sources describing designing playgrounds in (nursery) schools or day-care centers because they are not always accessible to citizens as well as books and book chapters because municipal design teams are unlikely to use them.

Identified sources from Google search.

The Google search generated 183 from 203 sources for being screened according to inclusion criteria screening. Records were excluded as 6 URLs could not be opened and 16 duplicates were removed.

The screening phased yielded 39 sources. Reasons for exclusion were the source was not about play (n= 6), the source was not about a playspace (n= 18), the source was not about a public playspace but about a playspace for example for a school or kindergarten (n=27) and the source was not about designing a public playspaces but for example about safety or not a guideline but a blog about a new designed playground (n=91).

Identified sources through search at websites of play organizations.

This screening of the five sources showed five documents eligible for assessment on children’s participation or an umbrella concept.

Identified sources through consultation of experts.

The 130 identified documents or URLs provided by experts yielded 94 records after removing 16 duplicates and 20 unopenable URLs for screening against the inclusion criteria.

This screening process resulted in 48 sources identified as a guideline for designing a public playspace. The exclusion reasons were 20 pages or URLs were not found, 16 duplicates were removed, 6 sources were not about play but addressing children’s health and well-being, 8 sources were not addressing a public playspace but a playspace at kindergarten or school and 32 sources could not be identified as a description for designing a playspace but were addressing other aspects about play in the community such as a strategy for improving play provision.

Selection phase

The selected sources from the screening phase, a total of 92 out of 3 search sources, were first checked for duplications. The remaining 76 sources were selected when children’s participation or parts of it as described in Article 12 of the United Nations Convention on the Rights of the Child, or umbrella concepts were addressed.

This process resulted in 42 guidelines for designing a public playspace that describe children's participation, community involvement or stakeholder consultation.

Supplement File S2: Additional information to Table 1 (A descriptive summary of the included guidelines)

No.	Affiliated institution or organization	Year of publication	Authors	Seize document	Topics addressed	Special focus	Reference to UN CRC	Assessment quality with ACE tool	URL
1	Australian Heart Foundation	2013	not described	40 pages	child-centered approach, project elements, project methodology, evaluation methodology, older children's voices, design process, site construction and evaluation and planning for active play	playspaces for older children, 8-12 years	Art 31	minor concerns	https://fdocuments.in/document/space-for-active-play-healthy-active-by-the-design-of-play-spaces-for-the.html?page=1
2	CABE	2008	not described	8 pages	state of play, planning for play, designing for play, back to nature and principles for designing play and case studies			serious concerns	https://www.designcouncil.org.uk/fileadmin/uploads/dc/Documents/designing-and-planning-for-play.pdf
3	Christopher and Dana Reeve Foundation	n.d.	not described	20 pages	inclusive playground, importance of play and build community pride	inclusive playspaces		serious concerns	www.lesliemccullough.com/uploads/5/0/7/0/5070598/playground_toolkit_final.pdf

4	City of Ballarat	2014	not described	33 pages	current issues in outdoor play, key directions and planning framework, design guidelines, universal access and inclusion, participation and challenges in play, community engagement and site practicalities and maintenance		serious concerns	https://www.ballarat.vic.gov.au/sites/default/files/2019-04/Play%20Space%20Planning%20Framework.pdf
5	Creo	n.d.	not described	11 pages	design, team building, formulating vision, planning for success, caring for the environment		serious concerns	https://creospace.co.nz/council/
6	Denver Parks and Recreation	2017	not described	79 pages	nature benefits, site selection, public engagement, inclusion in nature play, design and construction and case studies	nature play in the built environ ment	serious concerns	No URL available
7	DESSA	2007	not described	44 pages	the importance of play, how to make sure that disabled children can participate in play, consulting with children and planning and designing a playground	playspac es for disabled children	serious concerns	https://www.dessa.ie/wp-content/uploads/2021/03/Play-for-All.pdf
8	Free Play Network	2008	Aileen Shackler,	32 pages	10 principles for designing play spaces		serious concerns	www.freeplaynetwork.org.uk/designforplay/

			Nicola Butler, Phil Doyle and David Ball						
9	Geelong Australia	2012	Wendy Holland and Shaun Quayle	128 pages	planning and design for play spaces and play opportunities, management, marking and maintenance			moderate concerns	https://hdp-au-prod-app-ggc-yoursay-files.s3.ap-southeast-2.amazonaws.com/4316/0542/2931/8cf1e7654edc563-0220Play20Strategy20Part202.pdf
10	Government South Australia	n.d.	not described	51 pages	importance of inclusive play and connection through inclusive play	inclusive play and accessible playspaces		serious concerns	Inclusive Play - Guidelines for accessible playspaces - Easy Read
11	Greater London Authority	2012	Hermine Sanson, Jane Carlsen and Mike Newitt	106 pages	policy context, quality of a play space, neighborhood planning and accessibility			serious concerns	https://www.london.gov.uk/sites/default/files/osd31_shaping_neighbourhoods_play_and_informal_recreation_spg_high_res_7_0.pdf
12	Hags	2019	not described	56 pages	planning and preparation for a play space, play value and selecting play space equipment			serious concerns	https://issuu.com/hags-uk/docs/hags_-_inclusive_play_-_design_guid
13	HNH (Healthy New Hampshire)	2017	not described	66 pages	planning framework, resources, layout and how to select playground materials			serious concerns	https://issuu.com/hags-uk/docs/hags_-_inclusive_play_-_design_guid

	Foundation and NRPC (Nashua Regional Planning Commission)				and design considerations				
14	Illinois Department of Natural Resources	2004	not described	63 pages	definitions and terminology, planning considerations, site analysis, design and installation, maintenance and inspection			serious concerns	https://vdocument.in/a-guide-to-playground-planning.html?page=1
15	Inclusive SA (South Australia)	n.d.	not described	40 pages	inclusive play and connection with the place, each other and with self.	inclusive play and accessible playspaces		serious concerns	https://inclusive.sa.gov.au/__data/assets/pdf_file/0006/95460/3387-DHS-SA-Inclusive-play-case-studies-ER-FA-Accessible.pdf
16	Inspiring Scotland, Play Scotland, and the Nancy Ovens Trust	2018	Theresa Casey and Harry Harbottle	60 pages	key concepts and creating organized, accessible and inclusive design		Art 12<br>Art 23<br>Art 31<br>GC 17	moderate concerns	https://www.playscotland.org/play/playful-communities/free-play-guide/
17	Landcom	2008	not described	56 pages	design principles, delivery, ownership and maintenance			serious concerns	https://www.landcom.com.au/assets/Publications/Statement-of-Corporate-Intent/612d4e710e/open-space-design-guidelines-lr.pdf

18	Landscape Structures Inc.	2018	not described	20 pages	not described	inclusive playspaces		serious concerns	http://viewer.zmags.com/publication/c878a7ae#/c878a7ae/1
19	National Playing Fields Association	2004	John, A. and Wheway R.	45 pages	the Disability Discrimination Act, value of play and recommendations for local playground	playspaces for disabled children		minor concerns	www.childrensplayadvisoryservice.org.uk/pdf_files/Publications/can_play_will_play-CPASwebsite.pdf
20	NCB (National Children's Bureau)	2009	not described	16 pages	engagement of children and young people in the design process and practice examples	children and young people participation in design	Art 2 Art 12	serious concerns	No URL available
21	NSW (New South Wales) Government	2019	not described	78 pages	the inclusive play processes	inclusive playspaces		serious concerns	https://www.planning.nsw.gov.au/-/media/Files/DPE/Guidelines/everyone-can-play-guideline-2019-02-20.pdf
22	Office of Deputy Prime Minister	2003	Karen Dunn, Michele Moore and Pippa Murray	40 pages	understanding inclusive play design, engaging with disabled children and families and inclusion by design	accessible, inclusive playspaces	Art 31	moderate concerns	https://www.thenbs.com/PublicationIndex/documents/details?Pub=ODPM&DocID=265752
23	Play England, Department for Children, Schools and	2008	Aileen Shackell, Nicola Butler, Phil Doyle	156 pages	design specification and management issues for play		Art 31	serious concerns	https://www.playengland.org.uk/designforplay#:~:text=A%20guide%20to%20creating%20successful,experience%20risk%2C%20challenge%20and%20excitement.

	Families, Department for Culture, Media and Sport		and David Ball						
24	Play Wales	2012	not described	6 pages	local community groups, creating playable spaces, changing minds and changing spaces, repair and maintenance			serious concerns	www.playwales.org.uk/login/uploaded/documents/INFORMATION%20SHEETS/play%20spaces%20-%20planning%20and%20design.pdf
25	Play Wales	2016	not described	37 pages	design and management of play spaces		Art 12 Art 15 Art 31	serious concerns	https://issuu.com/playwales/docs/community_toolkit_2016
26	Play Wales	2021	not described	60 pages	design including participation, design, risk management and management with the mechanics of managing an existing or newly built play space		Art 12 Art 15 Art 31 GC 17	serious concerns	https://www.playwales.org.uk/eng/publications/communitytoolkit
27	Playcore	2012	not described	16 pages	creating a vision, identifying goals, engaging the community and ensuring play value			serious concerns	https://www.playandpark.com/uploads/wysiwyg/Blueprint-for-Play-Design-It.pdf
28	Playground Ideas	n.d.	Marcus Veerman	51 pages	Listen, plan, design, build and play		Art 31	serious concerns	https://www.playgroundideas.org/wp-content/uploads/5-step-Manual-.pdf
29	Playright	2016	Chris H.C. Yuen and	132 pages	relevance of inclusive play, design principles for inclusive play spaces, good practice,	inclusive playspaces	Art 2 Art 23 Art 30 Art 31	minor concerns	https://www.playright.org.hk/wp-content/uploads/2018/12/Playright-Inclusive-Play-Space-Guide.pdf

			Theresa Casey		universal design and indicators for inclusive playspaces				
30	Playworld	2015	not described	72 pages	playground standards, planning and preparation, play richness and selecting playground equipment.	inclusive playspaces		serious concerns	no URL available
31	Playworld Systems	2015	not described	13 pages	building a committee, locating a site and consulting a playground specialist and selection of playground equipment			serious concerns	https://playworld.com/psi_files/web/dwnld/Playground_101_Guide.pdf
32	Playworld Systems	2019	not described	45 pages	playground standards, planning and preparation of a playspace, play richness and selecting equipment.	inclusive playspaces	Art 31	serious concerns	https://response.playworld.com/inclusivedesignguide
33	Real Play Coalition	2020	not described	36 pages	the power of play and current issues, the urban framework and activating play.	playspaces in cities	Art 31	serious concerns	https://www.arup.com/perspectives/publications/research/section/reclaiming-play-in-cities
34	Rick Hansen Foundation	n.d.	not described	96 pages	creating an inclusive play space, best practices and common problems	inclusive playspaces		serious concerns	https://www.rickhansen.com/sites/default/files/downloads/letsplaytoolkit.pdf
35	Rick Hansen Foundation	2020	not described	40 pages	accessible play spaces, designing and building accessible play spaces, best practices and common issues,	accessible playspaces		serious concerns	https://www.rickhansen.com/sites/default/files/downloads/sch-35913-guide-creating-accessible-play-spaceswebaccessible.pdf

36	State of Victoria, Dept for Victorian Communities	2007	not described	80 pages	benefits of play, planning and providing accessible playspaces and design ideas		Art 12	serious concerns	https://www.playaustralia.org.au/sites/default/files/LibraryDownloads/The%20Good%20Play%20Space%20Guide%20I%20Can%20Play%20Too.pdf
37	Touched by Olivia	n.d.	not described	7 pages	everyone can play, connection to community, access to nature, play independence, friendship and social participation	inclusive playspaces		serious concerns	www.touchedbyolivia.com.au/wp-content/uploads/The-6-Principles-for-Inclusive-Play.pdf
38	Tualatin Hills Park & Recreation District	2012	S. Jo Linden and Bruce Barbarasch	24 pages	nature play and free play natural playscapes	nature play areas		serious concerns	www.touchedbyolivia.com.au/wp-content/uploads/The-6-Principles-for-Inclusive-Play.pdf
39	Unknown	2014	not described	113 pages	recreational trails and beach access routes and outdoor play spaces			serious concerns	https://www.cambridge.ca/en/learn-about/resources/DOPS-Guidelines.pdf
40	Waverley Council	2021	not described	81 pages	inclusive play context, strategic planning framework, Waverley context, community consultation and inclusive play space principles	inclusive playspace	Art 31	minor concerns	https://haveyoursay.waverley.nsw.gov.au/inclusive-play-space-study
41	Wexford County Council Communit	2018	not described	37 pages	team building, site identification, design and plan, organizing funds and installation			serious concerns	https://www.wexfordcoco.ie/sites/default/files/content/Community/Developing-a-Play-Area-in-your-Community-A-Step-by-Step-Guide-2018.-V.3.pdf

	y Developme nt Department				and management of a play area.			
42	Wokingha m Borough Council	2018	not described	62 pages	play space standards, design principles and inclusive design	Art 31	serious concerns	https://www.wokingham.gov.uk/council-and-meetings/open-data/plans-policies-and-strategies/?assetdet91f252ff-550d-4cfa-a838-92ef2cb5f83c=462172&categoryesctl91f252ff-550d-4cfa-a838-92ef2cb5f83c=2560&p=4

Legenda: UN Convention on the Rights of the Child:

Art 2 (non-discrimination)
Art 12 (sharing their views and given due weight)
Art 15 (assembling, to set up groups)
Art 23 (rights of children with disabilities)
Art 30 (children of minorities and indigenous groups)
Art 31 **(rest, leisure, play, recreational activities, cultural life and the arts)**
GC 17 (general comment for further understanding of Arti

Supplement File S3: Strategies for community involvement and children's participation represented in guidelines for designing public playspaces.

[x] refers to corresponding guideline, see table 1.

Theme	Strategies[1] for community involvement	Considerations, actions and plans for operationalizing the strategies for community involvement
Giving space and time for consulting with the local community.<br><br>34 references	Consulting with community members [9,16,27,21,23,35,39,42].<br><br>Consultation with the impacted community members (including businesses) and/or with the public.<br>Consider involvement of elected officials.	Communicate that a playspace should be seen as a space for the whole community.<br><br>Engage with user groups.<br>Engage with local children.<br>Engage with interested professionals.<br>Engage with members of local organizations: youth groups, church groups, schools and day-care centers, art groups, neighborhood organizations, vendors and business leaders, gardening club, public health, etc.<br>Engage with children with disabilities together with their informal or formal caregivers.<br>Engage with maintenance staff.<br>Use intermediaries when to access groups you are finding hard to reach,<br>Inform or involve elected council members of the area.<br>Consider who is missing in this consultation loop.<br>Engage with play equipment manufactures later in the process when a draft of the design has been discussed.<br><br>Consultation needs to be scaled with the size of the development.
	Consulting with users, local children, and young people [1,6,9,21,27,39,42].<br><br>The users can be children and young people of the neighborhood as well as from a wider distance. Children and young people can be approached through community-based children and youth groups or schools. Adult facilitators of youth groups and teachers might be enabling or hindering gatekeepers.	
	Consulting with children with disabilities and their caregivers [15,22,26, 27,37,39].<br><br>All children's voice should be considered and to create an inclusive playspace.<br>Children with disabilities and their caregivers can give valuable insights.	
	Consulting with professionals [15,16,21,22,35].	

[1] The word strategy is used in the meaning of "a way of doing something or dealing with something" (*Cambridge Dictionary,* n.d.)

	Think about officers/technicians from planning, design and maintenance as well as children's experts such as facilitators of youth groups, teachers and parents.	Community involvement starts at an early stage.<br>Reconnect with key groups periodically.<br>Involve the community in the planning, design, construction, maintenance and progressive enhancement phase.<br><br>All meetings but for sure the initial meeting should be interactive.<br><br>Communicate with officials and elected officers, all the people who take part in the community involvement, funders and local/national media.<br>Give feedback about the process and what has been done with their shared information on a regular base.
	Engaging early from the start [17,18].<br><br>Aligning with like-minded community members at the start.<br>Connect with all stakeholders early.	
	Consulting regularly throughout the process [6,11,17,22,28].<br><br>Go back to the community regularly, certainly at key stages such as planning, design, construction and maintenance.	
Identifying the needs of the community, beyond play, through an active, meaningful, and empowered approach.<br><br>43 references	Giving information about the project and processes [1,17,27,37].<br><br>Giving information about the planning and design process.<br>Giving information about the community engagement strategy.	Use an Assets Based Community Development approach instead of agenda of fixing problems.<br><br>Align yourself with like-minded community members.<br><br>Key is to get an insider perspective.<br><br>The steering group gives information about the project to the involved key stakeholders.<br>The steering group shares themes and ideas and asks for feedback.<br><br>Engage with the local community in a realistic and meaningful way:<br>- Use different sources: files and records, public hearings, surveys, children's classroom projects, staff, recommendations and professional consultants.<br>- Use collaborative approach.<br>- Be transparent.<br>- Be active and fully engaged.
	Providing site visits with informal engagements and discussions [11,14,23,27,28].<br><br>Identify needs of community, e.g., how place is used in existing conditions, level of services, access, benchmark to neighborhood, potential resources, needs and wishes of users and caregivers.	
	Connecting with users and community members through intermediaries, local forums, and families [16,22,27].<br><br>Local groups, professionals, volunteering groups and families can facilitate participation of children and young people.<br>Participation can be electronically facilitated.	
	Applying an actively and strength-based approach with community members and children and young people [2,3,4,5,6,7,10,13,14,21,22,27,28,37].	

	Consultation has to be realistic and meaningful.<br>It can draw on different formal and informal engagement sources e.g., public hearings, classroom/group activities, surveys, observations and the advisory board.<br>Participation needs a facilitator who can connect, communicate and positively interact with children and community.<br>Talk about what they are already doing at the space and about their wants and needs.<br>The process needs to be transparent and requires regularly update to reflect the changes in the project.	- Ask what community members want.<br>- Go out and ask what they are already doing.<br>- Listen to how the community already support their children.<br>- Identify spaces that will best serve their play priorities.<br>- Identify underutilized spaces.<br>- Organize informal engagement at the play space.<br>- Observe children and other people at the playspace at different times: how long do they spent time at the place, how is equipment used, what do people of different ages do.<br>- Identify wants and needs of different stakeholders.<br>- Set up design sessions, in any format, providing the listening, learning and imagining that makes your playspace come to life.<br>- Give considerations to the information shared by stakeholders.<br>- Be constantly prepared to revise attempts to engage with children and the community.<br>- Inform the stakeholders about the decisions to be made.<br><br>Be aware of changing needs of the community so set up a sustainable review process.<br><br>Children and young people should be asked every year how satisfied they are with the playspace.<br><br>Questions for community members<br>- Which experiences have you enjoyed when you were a child?<br>- What experiences do you see children enjoying now?<br>- What kind of experiences would you like to see children to be exposed to?

<table>
<tr><td></td><td></td><td></td></tr>
<tr><td rowspan="2">Establishing a shared vision responsive to community's needs.

64 references</td><td>Acknowledging children's expertise [1,4,11,13,16,20,21,22,25,27,33,39].

Children are the users and experts of a playspace.
Children's view and adult's view on what they want can differ.
The combined expertise of children and adults will yield better decisions.
To give children entitlement as community member.
To provide a genuinely children influenced playspace.
To value inclusive consultation.
To facilitate children's sense of empowerment and agency.
To raise children's self-confidence as community member.
To give children opportunities for learning about democracy and tolerance, for acquiring skills and knowledge about decision-making processes.</td><td rowspan="2">Children are experts of their own live and know what they want.
Children are not only the users of the playspace but also the creators.

What adults wants in a playspace may not be the same as what children want.
Some adults may have forgotten what it is like to be a child.

Community involvement provides the wider community an exposure to the project.
It brings an understanding of the lived experiences of the community residents.
Outcomes of the project will be more meaningful when a variety of stakeholders are involved.

To provide a genuine children influenced playspace.
To facilitate children's sense of empowerment and agency in their local community.
To ensure that outcomes are responsive to community needs and aspirations in current and future provision.
To provide valuable insights about diverse needs of children and caregivers.
To produce better council's services.
To get a better used playspace.
To provide public surveillance
To have a sense of ownership over the site.
To enhance the strategic approach at a local level through local play partnership.
To raise self-confidence of individuals and groups.
To affirm entitlement as community members and organizations.</td></tr>
<tr><td>Aiming for community's ownership and reduction of risks of vandalism [1,4,6,7,9,11,13,16,20,22,23,24,27,28,31,36,37].

To get a better used playspaces.
To get more informed decisions and improved designs.
To optimize effectiveness and utilization of the playspace and neighborhood.
More likely that the community uses playspace and provide public surveillance.
To achieve that the community (children and adults) takes ownership over the place.
To reduce risks of vandalism.
To create greater tolerance to outdoor play.
It will enhance a strategic approach to play at a local level.</td></tr>
</table>

	Achieving outcomes responsive to community's needs and aspirations. [2,4,6,8,11,20,27,29,33,36,40].<br><br>Meeting the needs of the community.<br>Consulting effectively with users produces better services.<br>Outcome is assured to be more meaningful.<br>Understanding what kind of activities children, young people and the community enjoy in their free time.<br>Funding is spent as good practice.<br>Getting liveable, enjoyable and sustainable environments for everyone.<br>Forming relationships between municipality and the community that can be beneficial to other projects.	Integrate playspaces into the framework of the existing neighborhood to optimize effectiveness and utilization.<br><br>Explore what is already known in the community about the space and about children's play.<br><br>Assure that the local community recognize priorities and expectations in the proposed design.<br><br>Community involvement and children's participation is often a condition of funding.
**Theme**	**Strategies[2] for children's participation**	**Considerations, actions and plans for operationalizing the strategies for children's participation**
Giving children safe, inclusive opportunities to form and express their views about playspaces.<br><br>101 references	Early involvement of children [1,6,11,16,26, 29,35,39,41].<br>10 references	Start early meetings and conversations as it can ultimately lead to a more cost-effective project.<br><br>Don't reach for the equipment catalogues but think creatively and broadly about the kinds of experience you hope the space will offer.<br><br>Early involvement of children can generate excitement.<br><br>Early participation in concept, design and implementation stages will result in increased ownership.<br><br>Create sustainable involvement through validation against other children in same age group, with the general community and with professionals.
	Creating sustainable involvement [1,6,11,12,20,21,22,26,27,29,30,36].<br>25 references	
	Ensuring an inclusive and accessible process [1,6,16, 19].<br>5 references	
	Involving those children who are affected [1,6,7,9,11,12,16,18,19,20,22,26,27,28,29,30,31,32,34,35,36,38,39,40,41].<br>63 references	
	Supporting children to feel safe and comfortable expressing themselves [1,6,27]. 3 references	
	Being able to provide support for when children become upset.<br>0 references	

[2] The word strategy is used in the meaning of "a way of doing something or dealing with something" (*Cambridge Dictionary,* n.d.)

		Arrange different meetings, e.g., town hall, on-site, design and constructing together meetings, which will facilitate sustainable involvement of children.<br><br>An ongoing consultation helps to understand changing priorities, cultural considerations and expectations and helps to establish relationships.<br><br>The best consultation processes are two-way processes that involve ongoing dialogue and mutual learning.<br><br>The participation meetings have an experiential learning component and a reflection and discussion component and invite each child in a dialogue.<br><br>Participation processes should involve children and young people, teachers, parents/caregivers and the local community.<br><br>The preferred approach is via knowledgeable facilitators who are open and can engage in dialogues.<br><br>The facilitator should share the purpose of the workshop and explain the ground rules for participation.<br><br>Stations for consultation should be set up in strategic locations.<br><br>Children can be involved in<br>- Basic inspections and reporting;<br>- Developing a survey;<br>- Expressing what they want and need;<br>- Sharing information about what they like and dislike about the site;

<table>
<tr>
<td></td>
<td></td>
<td>- Telling what may be missing in the current playspace;
- Advising and informing the design brief.

Make sure you talk directly to the children.

Additionally, listen and observe children playing at existing playgrounds.

Conduct targeted outreach to underrepresented members such as children with disabilities and their parents/caregivers.

Consultation with any hard-to-reach group takes time which should be built in the process.

Views should be collected of the children that make up the community, including variety in age, ability, gender, socio-economic status, race and culture.</td>
</tr>
<tr>
<td rowspan="6">Facilitating children to express their views.

113 references</td>
<td>Ensuring there is a list of topics on which you want to hear children's views [1,6,7,16,23,27,28,36,41].
18 references</td>
<td rowspan="6">Consultation and engagement activities require a plan:
- What do you need to find out?
- What are the hopes and aspirations for the play space?
- What ideas do people have for the design?
- What general character would people like the space to have?
- What do children and young people want to be able to do/experience?
- What do people need to make it more inclusive and accessible space?

Different ways of collecting children's views
- Drawing a picture of their playground;
- Drawing a map of their community and identify places where they have played;
- Model designs, e.g., with loose materials or plasticine;</td>
</tr>
<tr>
<td>Ensuring that the key focus of the process stays on the topic [20,41].
2 references</td>
</tr>
<tr>
<td>Informing children that participation is voluntary at any time
0 references</td>
</tr>
<tr>
<td>Supporting children in giving their own views [1,6,7,9,13,16,20, 22,23,25,26,27,28,34,35,36,39,42].
36 references</td>
</tr>
<tr>
<td>Ensuring a range of ways for children to express themselves [1,4,11,16,20,22,25,26,27,28,29,31, 34,35,36,41].
54 references</td>
</tr>
<tr>
<td>Allowing children to identify topics to discuss to add to list of topics [1,20,27].
3 references</td>
</tr>
</table>

		- Taking children to playgrounds outside their area;<br>- Discussions in small groups;<br>- Focus groups together with community members and recreation organizations;<br>- Art and design activities;<br>- Display plans and ask feedback;<br>- Go out and talk to children wherever they are;<br>- Drama and role-play;<br>- Photography;<br>- Video work;<br>- Visual and/or sensory mapping;<br>- Story telling;<br>- One-to-one or peer interview;<br>- Physical games;<br>- Show images of inclusive playspace to elicit a discussion;<br>- Questionnaire to seek feedback on a concept;<br>- Organized activities to experience the environment, e.g., bush walks, constructing sand sculpture or making a mud brick;<br>- Site visits or introduce temporary props and images;<br>- Ask children to teach you a game;<br>- Some resources developed children's participation purpose in designing a playspace, e.g., Pac-a-Map.<br><br>Questions to ask children:<br>- Where do you play?<br>- What do you do when you play?<br>- What do you like to play?<br>- What kind of spaces do you like?<br>- What features and activities are interesting you?<br>- What do your siblings like?<br>- What do you do as a family?

		- Are there any particular themes you think would suit the character of the space?<br>- What are joyful experiences?<br>- What are the most dangerous, scary places you have ever gone?<br>- Where would you like to alone?<br>- Where do you like to be with friends?<br>- What is the silliest thing you have ever done?<br>- What games do you invent?<br>- What activities would you like to try which you haven't tried before?<br>- What have you missed?<br><br>Better not ask children:<br>- What playground equipment would you like?<br><br>The facilitator should consider:<br>- Adapting the techniques to the needs and aspirations of the different ages, preferences and background of the children.<br>- Stretching children's imagination beyond their existing knowledge.<br>- Give children extra support, e.g., model-making workshop.<br>- Providing extra support and materials for children with special needs, e.g., for communication and for understanding.<br>- Leading discussions surrounding play, community, favorite activities and considerations such as inclusion, nature, colors, surfacing and maintenance.<br><br>A series of design considerations should be considered before narrowing it down to a list of key outcomes.

		Children (and other stakeholders) can be asked to vote for the most valuable proposal.
Informing children who will be listening to their views on playspaces.<br><br>15 references	Informing children to whom, how and when views will be communicated, demonstrating commitment to be informed and influenced by their views [1,4,5,20,23].<br>5 references	Be clear with children that they are not designers.<br>Inform children that their ideas will inform the designer prior to the site design beginning.<br><br>Explain to the children that meaningful participation will require a commitment across the stages of the design process and will take time.<br><br>Tell the children that the participation process is intended to have the best outcomes for children and to create a child-friendly environment.<br><br>Inform the children what you need help with and that the specific needs of user groups regarding play will inform all those involved in the design process.<br><br>Explain to the children that their views but also site characteristics, local development plans, demographic data, historical information, local community consultation and outcomes of events will inform the design.<br><br>Inform the children that all ideas will be valued as insights and documented, e.g., in a report, in a display, consultation book. These tools can be used for outreach, advertising and grand and fundraising appeals and to generate excitement.<br><br>Clarify to the children that their views will be reported to the steering group and handed to the open space planners and landscape architects working on the playspace development.
	Showing your commitment to be informed and influenced by their views [9,16,20,22,27,41].<br>6 references	
	Informing children about the identification of decision-makers.<br>0 references	
	Reporting back to the children about the decision-making process in a child-friendly way [1].<br>2 references	
	Giving children the opportunity to confirm their views.<br>0 reference	
	Giving children a role in communicating their views [6,27].<br>2 references	

Informing children of actions taken as a result of their shared views<br><br>30 references	Informing children about the scope of influence [1,4,6,9,16,20,23,27,35,40,41]. 12 references	Inform the children about the parameters of their involvement (e.g., reflecting planning principles, budget).<br>Explain children that principles will always be adapted to suit each playspace and the local community's needs.<br><br>Give a checklist to guide the process.<br><br>Inform the children about the overall aim: all children and young people should be able to use the same playspace, enjoy different times of play, play with others and share experiences (and not that every play need is met).<br><br>Tell the children how important their role in the participation process is.<br><br>Explain the children that the time between different phases can take long time.<br><br>Explain that the participation process has to be fair but will not avoid any disagreements.<br><br>Inform children and their caregivers about the deadline for consultation.<br><br>Describe to the children the role of the facilitator in relation to the decision-maker(s).<br><br>Explain the children that all suggestions will be considered, but realizing all ideas is not feasible.<br><br>Summarize children's ideas and all elements under consideration for the playspace design and explain how they relate to each other.
	Giving age-appropriate feedback during the design process [1,20].<br>2 references	
	Planning to make sure that children's views impact on decisions [1,4,13,23,27,29,41].<br>9 references	
	Giving children age-appropriated feedback on how their views were used [1,20].<br>5 references	
	Providing opportunities to evaluate the participation process [1,20].<br>2 references	

		Let the landscape architect or designer present the concept design to the children in a child friendly way, free of jargon, with acknowledgement of their input. Children should be given an opportunity to question the designers and the decisions that had been made.<br><br>The council shows support to the design process in ensuring effective outcomes for the children involved and the space emerging.<br><br>Let children help the contractor to construct the site, e.g., tree planting.<br><br>Celebrating the opening of a playspace with the children offering a public opportunity to recognize the contribution of involved children.<br><br>Inform the children about maintenance activities.<br><br>Give children the possibility to give feedback on the participation process.<br><br>Let the children share their experiences in using the playspace, as a start of a process of continual review.
